# Drug Exchange between Albumin Nanoparticles and Erythrocyte Membranes

**DOI:** 10.3390/nano9010047

**Published:** 2018-12-31

**Authors:** Bilyana Tacheva, Boyana Paarvanova, Ivan T. Ivanov, Boris Tenchov, Radostina Georgieva, Miroslav Karabaliev

**Affiliations:** 1 Department of Physics and Biophysics, Faculty of Medicine, Trakia University, 11 Armeiska, Stara 6000 Zagora, Bulgaria; bilyana.tacheva@trakia-uni.bg (B.T.); boyana.parvanova@trakia-uni.bg (B.P.); ivanov_it@gbg.bg (I.T.I.); radostina.georgieva@charite.de (R.G.); 2 Department of Medical Physics and Biophysics, Medical University–Sofia, 1431 Sofia, Bulgaria; btenchov@gmail.com; 3 Institute of Transfusion Medicine, Charité-Universitätsmedizin Berlin, Charitéplatz 1, 10117 Berlin, Germany

**Keywords:** bovine serum albumin nanoparticles, phenothiazine drugs, erythrocyte membranes, hemolytic assay

## Abstract

The effects of thioridazine (TDZ) and chlorpromazine (CPZ) and bovine serum albumin nanoparticles (BSA-NPs) on erythrocyte membranes have been investigated. Two kinds of hemolytic assays were used; hemolysis under hypotonic conditions and hemolysis in physiological conditions. Under hypotonic conditions for 50% hemolysis, both TDZ and CPZ have a biphasic effect on membranes; namely, stabilization at low concentrations and destabilization after reaching a critical concentration. In physiological conditions, there are other critical concentrations above which both drugs hemolyse the erythrocites. In each case, the critical concentrations of TDZ are lower than those of CPZ, which is consistent with the ratio of their partition coefficients. When BSA-NPs are added to the erythrocyte suspension simultaneously with the drugs, the critical concentrations increase for both drugs. The effect is due to the incorporation of a portion of drug substances into the BSA-nanoparticles, which consequently leads to the decrease of the active drug concentrations in the erythrocyte suspension medium. Similar values of the critical concentrations are found when the BSA-NPs are loaded with the drugs before their addition to the erythrocyte suspension in which case the events of the partition are: desorption of the drug from BSA-NPs, diffusion through the medium, and adsorption on erythrocyte membranes. This result suggests that the drugs are not influenced by the processes of adsorption and desorption onto and out of the BSA-NPs, and that the use of BSA-NPs as drug transporters would allow intravenous administration of higher doses of the drug without the risk of erythrocyte hemolysis.

## 1. Introduction

Albumin nanoparticles are among the promising new directions in the search for appropriate drug delivery systems. These particles combine the excellent biocompatibility of albumin and the benefits of the high inclusion capacity of nanoparticles. Albumin is generally known to be a carrier of hydrophobic molecules in plasma [1], as well as, to accumulate in tumors [2,3]. It can be internalized by the cells of various tissues and cell lines through an interaction with albumin-binding proteins and receptors, and subsequent endocytosis [2]. Thus, albumin-based delivery systems exploit the unique binding and transport properties of albumin with the obvious goal of improving penetration into the tumor tissues and accumulation of the hydrophobic drugs at the site of action [4]. In such a way, there is no need for toxic solvents or surfactants usually accompanying the application of poorly water-soluble drugs. In addition, the association with the albumin prevents some drugs from degradation [1,5] increasing their half-life in systemic circulation.

Albumin nanoparticles (NPs) can be prepared from human serum albumin (HSA) or bovine serum albumin (BSA) by a variety of techniques [6] among which the desolvation is one of the most exploited. Using the desolvation method, albumin-NPs can be synthesized with different predictable and reproducible sizes [7,8] (by controlling the BSA concentration, pH, ethanol concentration, NaCl content), or with different shapes by dimethyl sulfoxide (DMSO) addition during desolvation step [9]. Both size [10] and shape [9] have been considered to be of importance for cellular uptake.

Albumin-NPs have proven useful for loading with a variety of antitumor [4,11,12] and antiviral drugs [5,13]. HSA-NPs were also investigated as a nonviral vector for the delivery of plasmid DNA [14]. Albumin-coated NPs were investigated as possible carriers for insulin delivery [15] and for carrying antisense oligonucleotides for the treatment of viral infections or cancer [16]. An additional benefit is the fact that albumin-NPs have scavenging and antioxidant activities and could be additionally loaded with species having antioxidant activity on their own [17].

Albumin-NPs and albumin-coated NPs are promising candidates as a systems for oral [15,18], intravenous [4], or ocular drug delivery [19].

Interactions of drugs and biologically active substances with biomembranes are complex phenomena from a chemical or physicochemical point of view. Depending on the nature of the selected drug, the biomembrane represents the final site of action in some cases. However, more frequently, the drug–membrane interaction is only an intermediate step in the biological or toxic activity, affecting the degree of drug penetration and distribution in the cytoplasm before reaching a specific intracellular target. In other words, the binding and distribution of drug substances in cell membranes deserve to be studied and precisely characterized for both known and novel biologically active compounds.

The membrane of the red blood cell is a gold standard as a model for the investigation of the interactions of different active compounds and particulate systems with biological membranes. For example, hemolytic assays have been widely used to test the cytotoxicity of drugs [20,21,22], surfactants [23], phytoextracts [24], as well as, nanoparticles [25,26,27,28,29,30,31]. It has been shown that size, surface charge as well as specific functionalizations of the nanoparticles are important for their incorporation in the lipid bilayer, as well as for their interaction with the glycocalyx and membrane proteins [32,33,34].

Phenothiazines are a group of drugs classified as antipsychotics and neuroleptics. They are used to treat schizophrenia and psychosis. The most studied drug in this group is chlorpromazine (CPZ). In addition to blocking specific cellular receptors, some of the various effects of CPZ can be attributed to the amphiphilic nature of the drug. The tricyclic group of CPZ is hydrophobic and interacts with the hydrophobic phase of the lipid membrane, whereas the propylamine hydrocarbon tail of the drug is hydrophilic and interacts well with the membrane lipids’ polar heads [35]. Furthermore, at physiological pH levels, the propylamine chain of the drug is positively charged, which facilitates the interaction with the hydrophilic portion of the lipid bilayer. Since the inner side of the human red blood cell (RBC) membrane contains almost all anionic phospholipids [36], electrostatic attraction forces can capture CPZ on the inside of the membrane [37]. The preferential accumulation of CPZ expands the interior of the RBC membrane, forcing it to deform inwards and transforming the shape of the cells from discocytes to stomatocytes [37]. Theoretically, the availability of CPZ on the intracellular surface of the cell membrane will allow the drug to exert pharmacological effects by directly affecting intracellular processes such as signal transduction or intracellular transport. Other studies have shown that CPZ also interacts with protein components in the membrane [38], and, in particular, with spectrin [39,40], which can explain the stabilizing effect of CPZ on the mechanical properties of the erythrocyte membranes.

CPZ has been involved in several studies with albumin and albumin-NPs. It interacts with albumin changing its shape, size, and fluorescence [41]. It was also shown that CPZ acted as an inhibitor of albumin-NPs and albumin-coated NPs uptake by the cells, suggesting an active NPs transport by clathirin-mediated endocytosis [14,15].

Less research has been conducted on the interactions of thioridazine (TDZ), the second drug that has been chosen for the studies in the current work, with biomembranes. Its effects are similar to those of chlorpromazine [42], occurring at lower concentrations, which is tied to its stronger hydrophobicity and lower critical micelle concentration (CMC) [43].

The main objective of this work was to investigate the exchange of CPZ and TDZ between bovine serum albumin-NPs (BSA-NPs) and the erythrocyte membrane by monitoring their effects on membrane stability.

**Figure 1:**
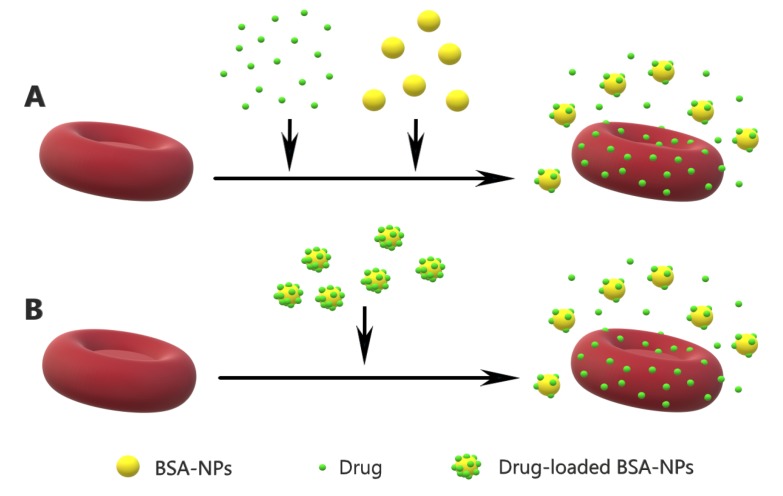
Shematic presentation of the hemolytic assays performed with simultaneously added drugs and BSA-NPs (**A**) and with drug-loaded BSA-NPs (**B**).

Two types of hemolytic assays were conducted; hemolytic assay under hypotonic conditions and hemolytic assay at physiological conditions. In both assays, the degree of hemolysis was determined by the optical absorbance of the hemoglobin released from the erythrocytes. The effects of the drugs on the hemolysis of human erythrocytes were investigated in the presence of the drugs alone, with simultaneously added drugs and BSA-NPs, as well as in the presence of drug-loaded BSA-NPs (Figure 1).

## 2. Materials and Methods

### 2.1. Drugs

The compounds, 2-Chloro-10-[3-(dimethylaminopropyl)] phenothiazine hydrochloride (Chlorpromazine) and (±) -2-methylthio-10-[2-(1-methyl-2-piperidyl) ethyl] phenothiazine hydrochloride (Thioridazine hydrochloride) are commercial products of Sigma-Aldrich, St. Louis, MO, USA. Solutions of 10 mM and 100 mM phenothiazine (chlorpromazine or thioridazine) were prepared in physiological saline (150 mM) of NaCl. Small volumes of these solutions were added to the experimental systems to achieve the desired concentrations.

### 2.2. BSA Nanoparticles

BSA nanoparticles (BSA-NPs) were obtained by the desolvation technique, a method previously described [44,45]. Briefly, 200 mg of Bovine serum albumin (BSA) (Sigma-Aldrich, USA) were dissolved in 2.0 mL of deionized water and the pH of the solution was adjusted to 7.4 with 0.01 M NaOH. Under constant stirring with 500 rpm, 8.0 mL of ethanol (Sigma-Aldrich, USA) was added to the initial albumin solution using a peristaltic pump at a rate of 0.5 mL/min. Consequently, the albumin molecules aggregated to multimolecular complexes with sizes in the nanoscale range. The obtained BSA-NPs were crosslinked by gradual addition of 0.2 mL of 8% glutaraldehyde solution (Sigma-Aldrich, USA) for the purpose of crosslinking the formed BSA particles during desolvation. After 24 h of incubation at 20 ${}^{\circ }$C with constant stirring, the NPs were purified by multiple washing steps with bi-distilled water and centrifugation at 14,500× *g*, and redispersed in deionized water at a final protein concentration of 10 mg/mL using ultrasound bath and stored at 4 ${}^{\circ }$C before use.

The size and zeta-potential of BSA-NPs were characterized by Zeta Potential Analyzer (NanoBrook 90Plus PALS, Brookhaven Instruments Corporation, Holtsville, NY, USA).

Drug-loaded BSA-NP were produced by incubation of a particle suspension (0.32 mg/mL protein in 149 mM NaCl and 5 mM phosphate buffer, pH 7.4 (PBS)) with different concentrations of the drugs (from 0.3 mM to 1 mM for TDZ and from 0.3 mM to 3.6 mM for CPZ, respectively) for 2.5 h at room temperature. After this incubation period the samples were centrifuged in order to separate the BSA-NPs loaded with the drugs from the surrounding medium in which there were still free molecules of the drugs. The loading of the drug in the BSA-NPs was estimated by measuring the difference in the concentration of the drug in the supernatant and the initial solution by spectrophotometry (absorbance of TDZ at 262 nm [46] and of CPZ at 254 nm [47,48]).

The drug release from the drug-loaded BSA-NPs was also characterized. For this assay drug-loaded BSA-NPs were dispersed in PBS to give concentrations of 0.32 mg/mL protein and ca. 165 μM TDZ or ca. 90 μM CPZ. Aliquots of these suspensions were transferred to centrifuge tubes. The tubes were kept at room temperature and centrifuged at 14,500× *g* at different time intervals (from 5 min to 95 min). The amount of the drugs released from the loaded BSA-NPs was determined in the supernatants by spectrophotometry, and converted to percentage from the initial drug amount.

### 2.3. Hemolytic Assays

Human erythrocytes were used as a model to study the interaction of the drugs and the BSA-NPs with biological membranes. Venous citrate blood was taken in a clinical laboratory on the day of investigation conducted with the informed consent of patients with normal blood counts and transmitted to the research team without the patient specific data. The blood was centrifuged at 1700× *g* for 4 min to separate the blood plasma and buffy coat. Erythrocytes were washed three times with physiological saline (154 mM NaCl) at a ten-fold volume dilution (1:10/*v*:*v*). A starting suspension of washed erythrocytes with a hematocrit of 20% was prepared.

#### 2.3.1. Hemolytic Assay under Hypotonic Conditions

During the hemolytic assay under hypotonic solution the starting cell suspension (75 μL) and a solution of the active ingredients with increasing concentration in each tube were added to 1.5 mL of 72 mM NaCl, 5 mM phosphate buffer pH 7.4 solution. This was an approximate concentration, at which a 50% hemolysis occurred. In the experiment with BSA-NPs equal volumes of 50 μL were added to each tube (final concentration of 0.32 mg/mL). The prepared tubes were incubated at room temperature for 30 min, then centrifuged for 2 min at 1700× *g* to remove the non-hemolyzed erythrocytes. The supernatant was diluted with distilled water at a volume ratio of 1:1 and the absorbance was recorded at λ = 555 nm (Spekol 11, Carl Zeiss, Jena, Germany).

The quantitative characterization of the drug’s effect is expressed by the relative absorbance (R.A.) [20,22].

(1) $$R.A.=\frac{A_{S}}{A_{50}}$$

Here, $A_{50}$ is the absorbance of the control sample with erythrocytes in a hypotonic solution for 50% hemolysis and $A_{S}$ is the absorbance of the sample with drug-treated erythrocytes under the same hypotonic conditions. The calculated R.A. takes values between −1 and 0, when the hemolysis is reduced by the drug, and values between 0 and 1, when the hemolysis increases in comparison with the control sample.

#### 2.3.2. Hemolytic Assay at Physiological Conditions

Durring the hemolytic assay under physiological conditions the erythrocytes were dissolved in a solution of 149 mM NaCl and 5 mM phosphate buffer, pH 7.4 (PBS). Different amounts of CPZ and TDZ were added to the suspension in order to find the concentrations that caused hemolysis at physiological conditions.

The effects of BSA-NPs were tested in two different type of experiments.

In the first type, different amounts of the drugs were added to the erythrocyte suspensions simultaneously with an equal amount of the BSA-NPs (final concentration of the BSA-NPs of 0.32 mg/mL). The obtained suspension was incubated for 30 min, and then centrifuged at 1700× *g*. The absorption spectrum of the supernatant was measured and the degree of hemolysis was determined by the absorption peak of the released oxyhemoglobin at 415 nm [49].

In the second type of experiment, BSA-NPs loaded with different amounts of the drugs were diluted with phosphate buffer to 1500 μL and this solution was kept for 30 min. In all samples, the final amount of BSA-NPs was 0.32 mg/mL. After 30 min the erythrocytes were added and the obtained suspension was incubated for another 30 min. Then the suspension was centrifuged and the amount of the released hemoglobin was determined in the supernatant by spectrophotometry at wavelength of 415 nm. The spectrophotometer used in these experiments was Cary 60 UV-Vis, Agilent Technolgies.

The results are expressed by the quantity relative hemolysis (R.H.), that is estimated by the equation [20,22]:

(2) $$R.H.=\frac{A_{S}-A_{c1}}{A_{c2}-A_{c1}}$$

where $A_{S}$ is the absorbance of the sample, $A_{c1}$ is the mechanical hemolysis control absorbance (no added drugs, erythrocytes in physiological buffered solution), and $A_{c2}$ is the 100% hemolysis control absorbance (erythrocytes in water).

## 3. Results and Discussion

### 3.1. Characterization of BSA-NPs

The size and zeta-potential of the synthesized BSA-NPs are determined by dynamic light scattering methods (Table 1). The measurements are performed in water and in PBS in order to test the suspension stability. The measured mean hydrodynamic diameter is 250 nm in water and 213 nm in PBS. Xie et al. [45] obtained similar values for the size of BSA-NPs fabricated by the same procedure and measured in water (214 nm). In PBS, they reported a value of 634 nm but explained the size increase by a formation of small aggregates in their samples during the measurement which was not the case in our samples. The different size of our BSA-NPs measured in water and in PBS could be due to conformational changes of the protein molecules. In water the protein molecules unfold due to stronger electrostatic repulsion forces between the mainly negatively charged functional groups. These charges are screened in the presence of small counterions and the protein molecules are more tightly folded. As a result, the particles slightly swell in water or shrink in PBS, respectively.

The measured values for the zeta-potential of the BSA-NPs are significantly lower in PBS compared to these in water also due to screening of the surface charges of the particles by counterions.

### 3.2. Loading Efficiency of CPZ and TDZ into BSA-NPs and Release Profiles

Approximately 48% of the initial concentration of TDZ and approxmitaley 27% of the initial concentration of CPZ are loaded into the BSA-NPs. When the BSA-NPs are incubated with 340 μM of the drug, this corresponds to an absolute amount of 201 μg TDZ and 54 μg CPZ per mg protein loaded into the BSA-NPs. Both drugs have a low solubility with cationic dissociation in aqueous solutions, which suggests both electrostatic and hydrophobic interaction to be responsible for their incorporation into the BSA-NPs.

**Figure 2:**
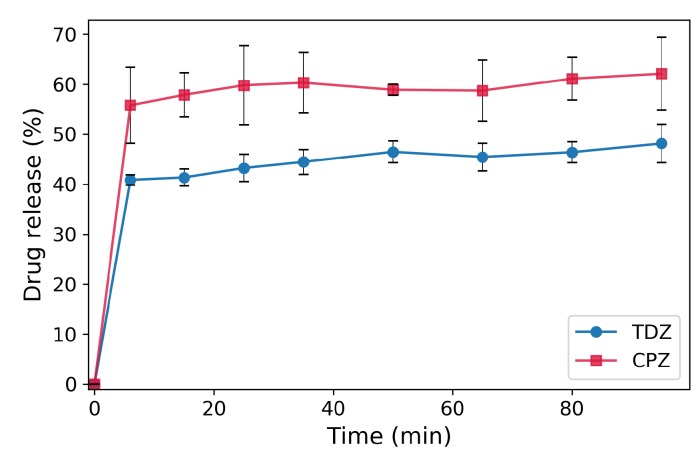
Release profiles of TDZ and CPZ from loaded BSA-NPs, presented as percentage of the initial amount of loaded drug.

The drug release measurements are performed at approximately 1% volume concentration of the loaded BSA-NPs, in order to assure conditions far from the saturation concentrations of the drugs (Figure 2). Both drugs show similar bi-phasic release profiles from the loaded BSA-NPs with a very fast burst immediately after immersing the particles in PBS and a slow release thereafter. The burst takes place within the first few minutes and results in 41% of the loaded amount of TDZ and 56% of the loaded amount of CPZ. The released amounts of the drugs increase to 48% and 62% for TDZ and CPZ, respectively, during the next 90 min. Most probably, the drug adsorbed on and near the particle surface is easily dissociated and released due to the high concentration gradient. Additionally, also some free drug is probably transferred together with the particle sediment into the release solution. The dissociation and release of the drug molecules incorporated deeper in the BSA-NPs are more difficult because they can undergo multiple interactions with the surrounding protein molecules, causing the second phase of slow release.

### 3.3. Effects of Drugs and BSA-NPs on Hemolysis in Hypotonic Conditions

The drugs’ effects on erythrocyte membranes are characterized in terms of induced changes in membrane stability against hypotonic hemolysis, which is the so called hemolytic assay under hypotonic conditions. During the assay erythrocytes are incubated in hypotonic solution with osmolarity that causes 50% hemolysis. When a drug is added to such suspension it could either stabilize or destabilize the erythrocyte membrane, resulting in decreased or incresed rate of hemolysis, respectively.

**Figure 3:**
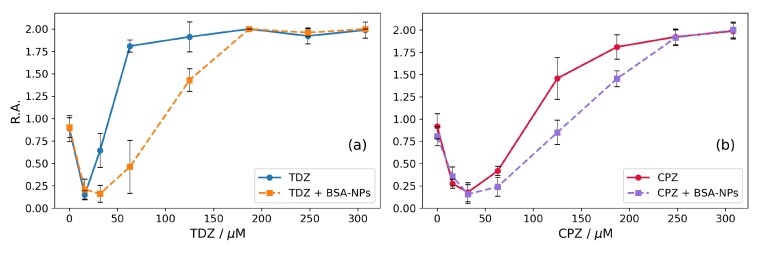
Influence of the drugs and combined influence of drugs and BSA-NPs on hypotonic hemolysis of erythrocytes (hematocrit 0.95%, 66 mM NaCl and 5 mM phosphate buffer, pH 7.4). Incubation time 30 min. The results are averaged over three measurements for each concentration of the respective drug substance. (**a**) TDZ (solid curve) and TDZ plus 0.32 mg/mL BSA-NPs (dashed curve). (**b**) CPZ (solid curve) and CPZ plus 0.32 mg/mL BSA-NPs (dashed curve).

The results for the action of TDZ and CPZ on erythrocytes are shown in Figure 3a and Figure 3b, respectively. Along with these, parallel measurements with the same amounts of drugs, but in the presence of BSA-NPs in solution have been performed and the results are shown in the same Figure 3.

Both phenothiazine derivatives show biphasic effects on the membranes (the solid curves in Figure 3a,b). At low concentrations, both CPZ and TDZ stabilize erythrocyte membranes and increase osmotic resistance against hypotonic lysis. Both solid curves in Figure 3a,b show decreased values of the relative absorbance (R.A. < 1) which means reduced hemolysis at low concentrations. At certain concentrations of CPZ and TDZ the opposite effect is observed, and the erythrocyte membranes are destabilized—the curves go upwards to R.A. values higher than 1. Finally, at specific concentrations of CPZ and TDZ, the erythrocytes hemolyse completely (R.A. = 2, corresponding to 100% hemolysis). The maximum protective effect is obtained at 16 μM for TDZ and 32 μM for CPZ, respectively, with CPZ stabilizing the erythrocytes in a larger range of concentrations up to 95 μM. For TDZ the stabilizing effect disappears already at 35 μM. Obviously, the effects are about 2–3 times stronger for TDZ, than for CPZ (i.e., the same effects occur at 2–3 times lower concentrations for TDZ). It is interesting to note that this result correlates with the ratio of the partition coefficients of these two drugs in liposomes $K_{p}^{TDZ}$/$K_{p}^{CPZ}$ = 2.22, as calculated from data reported by [42].

Previously, we have shown that BSA-nanoparticles can effectively incorporate drugs, in particular CPZ and TDZ [50]. Here, we investigate the effect of drug-loaded BSA-NPs on the membranes applying the hypotonic hemolysis of erythrocytes. The results are shown as dashed curves in Figure 3a,b.

Qualitatively, the effects are the same as in the case of the two drugs without BSA-NPs in the suspension. However, the presence of BSA-NPs in the suspension leads to a shift of the effects to higher concentrations for both TDZ and CPZ. Considering that BSA-NPs can effectively incorporate TDZ and CPZ [50], the results presented in Figure 3 can be interpreted as follows: The drugs are partitioned between the membranes and the BSA-NPs and the BSA-NPs can carry the drug to the erythrocyte membranes. Since the drugs are amphiphilic, they tend to dissolve in both the particles and the membrane. In this way, they are partitioned between the particles and the erythrocyte membranes in the solution. Accordingly, the effective drug concentration acting on erythrocytes is reduced due to the fact that part of the drug remains incorporated in the BSA-NPs. The effective concentration depends on the partition coefficients between the respective phases. According to our previous work [50] BSA-NPs incorporate 1.75 times more TDZ than CPZ as measured by the electrochemical method Cyclic Voltammetry—a value that is very close to the determined here value of 1.78 times (48% TDZ loading vs. 27% CPZ loading). It can be seen in Figure 3 that the lowest destabilizing concentration of CPZ (R.A. returns to 1, i.e., to 50% hemolysis) increases 1.47 times from 95 μM to 140 μM. For TDZ the required destabilizing concentration increases 2.71 times from 35 μM to 95 μM (Figure 3). Consequently, the presence of BSA-NPs affects the influence of TDZ on the hypotonic hemolysis (1.84-fold stronger than that of CPZ). The value of 1.84 which is obtained here is very close to the value of 1.75 for the ratio of incorporation of TDZ and CPZ into BSA-NPs obtained by Cyclic Voltammetry [50]. The similarity of these values, which are obtained by two independent methods, supports additionally the conclusion that the increase of the destabilizing TDZ and CPZ concentrations in the presence of BSA-NPs is due to the partial incorporation of the drugs into the BSA-NPs, i.e. partitioning of TDZ and CPZ between erythrocyte membranes and BSA-NPs. Accordingly, the hydrophobic interaction is the most probable mechanism of incorporation of the two drugs in the BSA-NPs.

### 3.4. Effects of Drugs and BSA-NPs on Hemolysis in Physiological Conditions

**Figure 4:**
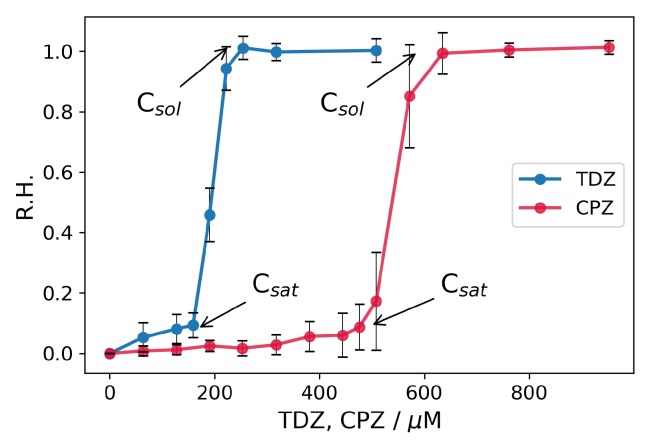
Relative hemolysis (R.H.) caused by the action of TDZ and CPZ on erythrocytes at physiological conditions (hematocrit 0.95%, 149 mM NaCl, 5 mM phosphate buffer, pH 7.4). Incubation time 30 min. The results are averaged over three measurements for each concentration of the respective drug substance.

In physiological conditions, the drug-induced hemolysis is due only to the interaction of the drug with the membrane. The results for TDZ and CPZ actions in physiological conditions are shown in Figure 4.

As can be seen in Figure 4, there is a threshold action of both TDZ and CPZ concerning the hemolysis of erythrosytes. Bellow this concentration, the drugs are adsorbed on the erythrocytes membranes without causing hemolysis. Above this concentration denoted as $C_{\mathrm{sat}}$ [21,23], the erythrocytes start to hemolyse extensively, and above some value $C_{\mathrm{sol}}$ are completely solubilised. The results for the threshold saturation concentrations are $C_{\mathrm{sat}}^{TDZ}\approx 160\;\mu$M for TDZ and $C_{\mathrm{sat}}^{CPZ}\approx 500\;\mu$M for CPZ. The obtained solubilsation concentrations are $C_{\mathrm{sol}}^{TDZ}\approx 220\;\mu$M for TDZ and $C_{\mathrm{sol}}^{CPZ}\approx 590\;\mu$M for CPZ. These values suggest 2.62 to 3.1 times stronger effect for TDZ, than for CPZ (i.e., the same effects occur at 2.62–3.1 times lower concentrations for TDZ), that is in correspondence with the effects found in the previous section with the hypotonic hemolytic assay.

**Figure 5:**
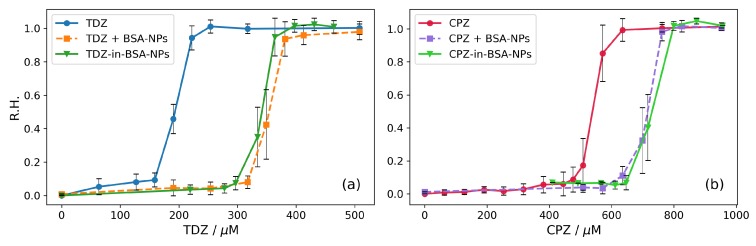
Influence of the drugs and combined influence of drugs and BSA-NPs on the hemolysis of erythrocytes in physiological conditions (hematocrit 0.95%, 149 mM NaCl and 5 mM sodium phosphate, pH 7.4). Incubation time 30 min. (**a**) TDZ alone (circles, solid blue curve), TDZ plus 0.32 mg/mL BSA-NPs (squares, dashed orange curve), 0.32 mg/mL BSA-NPs loaded with TDZ (triangles, solid green curve. (**b**) CPZ alone (circles, solid red curve); CPZ plus 0.32 mg/mL BSA-NPs (squares, dashed purple curve), 0.32 mg/mL BSA-NPs loaded with CPZ (triangles, solid green curve). The results are averaged over three measurements for each concentration of the respective drug substance.

The addition of BSA-NPs along with the drugs shifts the threshold concentrations to bigger values. The explanation is the same as in the case with hypotonic hemolysis. Part of the drug is absorbed in the BSA-NPs that decreases the concentration of the free drug’s molecules in the medium. The obtained results are shown in Figure 5. Besides the experiments with the simultaneous, addition of drug and BSA-NPs, parallel experiments are performed in which the drugs were previously absorbed in the BSA-NPs and the so-obtained drug-loaded nanoparticles are added to the erythrocytes. This is done to change the order of the interaction events in the three-phase system of erythrocytes–medium–nanoparticles. When the drug and the BSA-NPs are added simultaneously to the erythrocyte suspension, the drug starts to be adsorbed concurrently on the BSA-NPs and the erythrocytes. When the drug is previously incorporated into the BSA-NPs and the drug-loaded nanoparticles are added to the erythrocyte suspension the order of events is: desorption of the drug from the nanoparticle, diffusion through the medium, adsorption on the erythrocytes’ membranes. Both cases are represented in Figure 5.

It is obvious from Figure 5 that for both TDZ and CPZ, the presence of BSA-NPs shifts the concentrations for the threshold actions to greater values. The other thing to be pointed out is that the curves for simultaneously added drugs and BSA-NPs (dashed curves) and the curves for previously drug-loaded BSA-NPs (solid green curves) coincide for both TDZ and CPZ within the limits of the errors. This suggests that, in both cases, the action of the drugs on the erythrocytes’ membranes depends on its partitioning between the three phases in the system; BSA-NPs, medium and erythrocytes.

In the presence of 0.32 mg/mL BSA-NPs the threshold values for the onset of the hemolysis are for TDZ $C_{sat}^{TDZ(+BSA-NPs)}\approx 317\;\mu$M when TDZ and BSA-NPs are simultaneously added, and $C_{sat}^{TDZ(-in-BSA-NPs)}\approx 300\;\mu$M when TDZ is previously loaded in BSA-NPs. Compared to the value for TDZ alone which is $C_{sat}^{TDZ}\approx 160\;\mu$M the values for $C_{sat}$ with the BSA-NPs are in average 1.93 ± 0.06 times bigger. The values for the complete solubilisation $C_{sol}$ are $C_{sol}^{TDZ(+BSA-NPs)}\approx 380\;\mu$M and $C_{sol}^{TDZ(-in-BSA-NPs)}\approx 364\;\mu$M. Compared to the value for TDZ alone which is $C_{sol}^{TDZ}\approx 220\;\mu$M the values for $C_{sol}$ with the BSA-NPs are in average 1.69 ± 0.04 times bigger. This gives in average 1.81 times bigger concentrations for the same effects when BSA-NPs are present in the solution.

It is interesting to compare these values taking into account the estimated data for the partitioning of the drug between the BSA-NPs and the surrounding medium (TDZ is partitioned 48% in the BSA-NPs and 52% in the medium). According to this partition, at $C_{sat}^{TDZ(-in-BSA-NPs)}$ when the overall TDZ concentration is 300 μM the concentration of the free TDZ in the medium is estimated to be 156 μM, which is almost the same as $C_{sat}^{TDZ}\approx 160\;\mu$M. At $C_{sol}^{TDZ(+BSA-NPs)}$ when the overall TDZ concentration is 380 μM the concentration of the free TDZ in the medium is estimated to be 198 μM, a value which is comparable to $C_{sol}^{TDZ}\approx 220\;\mu$M.

For CPZ (Figure 5b) the values for the onset of hemolysis are as follows: $C_{\mathrm{sat}}^{CPZ(alone)}\approx 500\;\mu$M, $C_{\mathrm{sat}}^{CPZ(+BSA-NPs)}\approx 635\;\mu$M and $C_{\mathrm{sat}}^{CPZ(-in-BSA-NPs)}\approx 650\;\mu$M. For the values for complete hemolysis the results are: $C_{\mathrm{sol}}^{CPZ(alone)}\approx 590\;\mu$M, $C_{\mathrm{sol}}^{CPZ(+BSA-NPs)}\approx 760\;\mu$M and $C_{\mathrm{sol}}^{CPZ(-in-BSA-NPs)}\approx 800\;\mu$M. The threshold concentrations in the presence of BSA-NPs are shifted to greater values—in average 1.28 ± 0.02 times greater for $C_{\mathrm{sat}}$ and 1.32 ± 0.04 times greater for $C_{\mathrm{sol}}$. The values for the partition of CPZ between BSA-NPs and the medium obtained from the centrifugation assays are 27% of the drug in the BSA-NPs and 73% in the medium. Using the value of 73% we can estimate the free concentrations of CPZ for the cases with BSA-NPs in solution: approx. 475 μM for $C_{\mathrm{sat}}$ and 580 μM for $C_{\mathrm{sol}}$, which are pretty close to the values when CPZ is alone in the solution.

**Figure 6:**
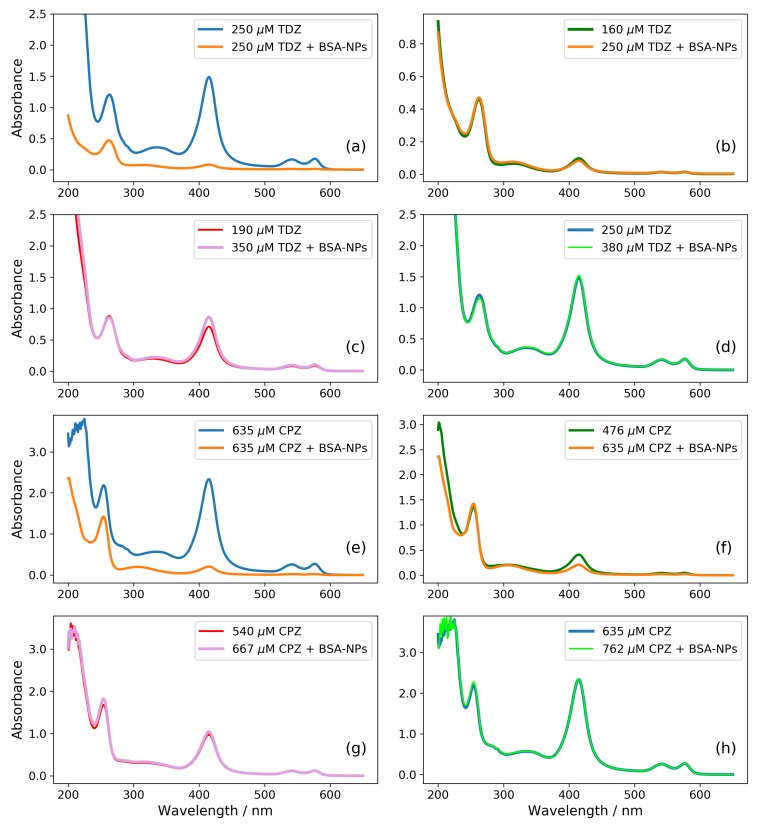
Absorption spectra of supernatants of erythrocytes suspensions with different amount of TDZ (**a**–**d**) and CPZ (**e**–**h**) without BSA-NPs or with 0.32 mg/mL BSA-NPs: Equal amount of TDZ (**a**) and of CPZ (**e**), provoking hemolysis without BSA-NPs and not provoking hemolysis in the presence of BSA-NPs. Different amount of TDZ (**b**) and CPZ (**f**) without and with BSA-NPs, both below the threshold concentration $C_{\mathrm{sat}}$ for the onset of hemolysis. Different amount of TDZ (**c**) and CPZ (**g**) without and with BSA-NPs, both above $C_{\mathrm{sat}}$ and below $C_{\mathrm{sol}}$. Different amount of TDZ (**d**) and CPZ (**h**) without and with BSA-NPs, both above $C_{\mathrm{sol}}$ for complete hemolysis.

An additional proof that the concentration of the free drug in the medium solution is essential for the observed effects on the erythrocytes membrane is presented in Figure 6. It represents absorption spectra of the supernatants obtained after 30 min of incubation with different concentrations of TDZ with and without BSA-NPs (Figure 6a–d) and with different concentrations of CPZ with and without BSA-NPs (Figure 6e–h).

In Figure 6a, we show two spectra obtained with same amount of 250 μM TDZ without and with BSA-NPs in solution. This concentration is above $C_{\mathrm{sol}}^{CPZ(alone)}\approx 220\;\mu$M when the TDZ is alone in the solution, but below $C_{\mathrm{sat}}^{CPZ(+BSA-NPs)}\approx 300\;\mu$M. Accordingly, this amount of TDZ completely solubilises the membrane and the hemolysis is 100% when there is no BSA-NPs (the blue curve in Figure 6a), but when there is BSA-NPs in solution, the percentage of the hemolysis is negligible. The hemolysis is expressed by the peaks of oxhyhemoglobin at 415 nm [49]. The spectral peaks of TDZ are at a wavelength of 262 nm [46]. In Figure 6a, one can see that the peak of TDZ (262 nm) is smaller for the case when BSA-NPs are present in solution (the orange curve). This suggests that the effective concentration of TDZ in the medium in the presence of BSA-NPs is smaller, and it is not big enough to provoke hemolysis. The remaining amount of TDZ is not in the supernatant but in the sediment together with the BSA-NPs. The spectra in the next three subfigures (Figure 6b–d) are chosen in such a way that they show equivalent hemolysis without and with BSA-NPs. In Figure 6b, both concentrations are bellow the corresponding $C_{\mathrm{sat}}$. In Figure 6c, both concentrations are between the corresponding $C_{\mathrm{sat}}$ and $C_{\mathrm{sol}}$. In Figure 6d, both concentrations are above the corresponding $C_{\mathrm{sol}}$. It can be seen, in all three figures (b, c and d), that the peaks of the released hemoglobin (at 415 nm) coincide, but in all three cases, the peaks of TDZ (at 262 nm) coincide too, despite the different initial concentration of TDZ (shown in the figure legends). This suggests that while the overall drug concentrations are different, equal amounts of the free drug in the solution medium provoke equal amount of hemolysis.

The same behavior is observed for the samples treated with corresponding concentrations of CPZ (Figure 6e–h). Here, the effects start at a higher concentration as already observed in Figure 5b due to the lower hydrophobicity of this drug compared to TDZ, which leads to incorporation of smaller amount into the erythrocite membrane.

## 4. Conclusions

The presence of BSA-NPs in a solution with suspended red blood cells leads to an increase in the critical concentrations of both CPZ and TDZ which destabilize the cells. The effect is expressed both in physiological conditions and under hypotonic conditions. The increase of critical concentrations is due to the incorporation of some of the drugs molecules into the nanoparticles and, hence, to a decrease in the free concentration of the drugs in solution. The partition of the drugs between the BSA-NPs and erythrocyte membranes is the same in the two investigated cases; when the drug is concurrently absorbed by BSA-NPs and membranes, and when the drug is previously loaded in BSA-NPs and the events of the partition are: dessorption of the drug from BSA-NPs, diffusion through the medium, and adsorption on erythrocyte membranes. This result also suggests that the drugs are not influenced by the process of adsorption and desorption onto and out of the BSA-NPs. The use of BSA-NPs as a drug transporter would allow intravenous administration of higher doses of the drug without the risk of erythrocyte hemolysis. The method used also allows a relative comparison of the partition coefficients between erythrocyte membranes, electrolyte solution and nanoparticles.

## Figures and Tables

**Table 1:** Size and zeta-potential of BSA-NPs. (In paranthesis are the polydispersity indexes.)

Suspension Medium	Mean Diameter (nm)	Zeta-Potential (mV)
water	250 (0.273)	−15.2±0.6
PBS	213 (0.295)	−5.4±0.2

## Abbreviations

The following abbreviations are used in this manuscript:

NPs	Nanoparticles
BSA-NPs	Bovine serum albumin nanoparticles
CPZ	Chlorpromazine
TDZ	Thioridazine
